# Diabetes among Ethiopian Immigrants to Israel: Exploring the Effects of Migration and Ethnicity on Diabetes Risk

**DOI:** 10.1371/journal.pone.0157354

**Published:** 2016-06-14

**Authors:** Anat Jaffe, Shmuel Giveon, Liat Wulffhart, Bernice Oberman, Laurence Freedman, Arnona Ziv, Ofra Kalter-Leibovici

**Affiliations:** 1 Endocrinology & Diabetes Unit Hillel Yaffe Medical Center, Hadera, Israel; 2 Clalit Health Services, Department of Family Practice, Sharon Shomron District, Department of Family Practice, Sackler School of Medicine, Tel-Aviv University, Tel-Aviv, Israel; 3 Unit of Biostatistics; Gertner Institute for Epidemiology & Health Policy Research, Sheba Medical Center, Tel-Hashomer, Ramat Gan, Israel; 4 Unit of Information and Computing, Gertner Institute for Epidemiology & Health Policy Research, Sheba Medical Center, Tel-Hashomer, Ramat Gan, Israel; 5 Unit of Cardiovascular Epidemiology, Gertner Institute for Epidemiology & Health Policy Research, Sheba Medical Center, Tel-Hashomer, Ramat Gan, Israel; 6 Sackler Faculty of Medicine, Tel-Aviv University, Tel-Aviv, Israel; Tel Aviv Sourasky Medical Center, ISRAEL

## Abstract

**Objective:**

Diabetes prevalence among ethnic minorities and immigrants often differs from the majority indigenous population. We compared diabetes prevalence, incidence and risk among Ethiopian and non-Ethiopian Jews. Within these main groups, we controlled for the effect of migration on diabetes risk by comparing the subgroups of Ethiopian and former Soviet Union (FSU) immigrants, and compared both with Israeli-born non-Ethiopian Jews.

**Methods:**

The study cohort included adult Ethiopian (n = 8,398) and age-matched non-Ethiopian Jews (n = 15,977) and subgroups: Ethiopian immigrants (n = 7,994), FSU immigrants (n = 1,541) and Israeli-born non-Ethiopian Jews (n = 10,828). Diabetes prevalence, annual incidence, and hazard ratios (HRs) adjusted for sex and metabolic syndrome (MetS)-components, were determined in three age groups (<50yrs, 50-59yrs, and ≥60yrs). Comparisons of body mass index (BMI) at diabetes incidence were made.

**Results:**

Younger (<50yrs) Ethiopians had higher prevalence rates, 3.6% (95%CI: 3.1–4.1) and annual incidence, 0.9% (95%CI: 0.8–1.0) than non-Ethiopians, 2.7% (95%CI: 2.3–3.0) and 0.5% (95%CI: 0.4–0.6), respectively. These differences were particularly pronounced among Ethiopian women. Diabetes risk among Ethiopians was higher and adjustment for MetS-components was important only for BMI, which further increased hazard ratio (HR) estimates associated with Ethiopian ethnicity from 1.81 (95% CI:1.50–2.17) to 2.31 (95% CI:1.91–2.79). The same differences were seen when comparing Ethiopian to FSU immigrants. BMI before incident diabetes was lower among younger Ethiopian immigrants than younger FSU immigrants and Israeli-born.

**Conclusions:**

Ethiopian ethnicity is associated with increased diabetes risk, which is age and BMI dependent. Young Ethiopians<50yrs, particularly women, had the greatest increase in risk. Lower BMI cut-offs should be defined to reflect diabetes risk among Ethiopians.

## Introduction

The emerging global epidemic of diabetes disproportionally affects indigenous and immigrant populations of non-European origin [1]. For example, immigrants from sub-Saharan Africa to developed countries tend to have a higher prevalence of diabetes than indigenous populations [1–4]. However, indigenous populations that preserve their traditional lifestyle are less affected; this is the case in rural Ethiopia where there is a diabetes prevalence of 1% [5–6].

Israel has absorbed many immigrant populations over the last century, most recently, Jewish immigrants from rural Ethiopia and from the former Soviet Union (FSU). Mounting evidence suggests that in non-European immigrant populations, transition from low to high diabetes prevalence occurs gradually, as has been documented among Jews who migrated to Israel from Yemen [7–8]. Diabetes was practically nonexistent among Ethiopian Jews upon immigration to Israel, but an increase in diabetes cases [9–10] associated with an increase in body fat and hypertension, has since been reported [11–14]. A recent cross-sectional study showed that the increase in diabetes and hypertension prevalence among Ethiopian immigrants to Israel correlated with a longer period of residence in Israel [15]. Nevertheless, most of these observed trends were based on small cross-sectional studies, and were not adjusted for age, sex or diabetes risk factors. Furthermore, cohort studies on diabetes incidence in recent immigrant populations to Israel have not been published.

Data from the second largest health fund in Israel show that the prevalence of diabetes among adult FSU immigrants is higher than among non-immigrant subjects, 10.1% versus 6.7%, respectively [16]. The greater diabetes risk among immigrant groups may be attributed to migration and acculturation stress. On the other hand, some ethnic groups may have higher susceptibility to diabetes, which becomes evident when they change their traditional lifestyle and environment [1, 15]. The objectives of this study were to determine prevalence, incidence and risk for diabetes among Ethiopian Jews and non-Ethiopian Jews based on a large cohort and to further study the combined effect of migration and ethnicity on diabetes risk, by comparing immigrants from Ethiopia and the FSU with Jews born in Israel. Diabetes incidence in these communities was also studied in relation to the components of the metabolic syndrome (MetS).

## Subjects and Methods

This is a historical-prospective cohort study. The study cohort included residents of the mostly urban Sharon-Shomron area in central Israel who were insured by Clalit Health Services (CHS), the largest health fund in Israel. CHS insures more than 86% of the Ethiopian Jews and 46% of the non-Ethiopian Jews in that district. Patient-related information was derived from the electronic medical records and the CHS administrative database. The database includes a list of all diagnoses, demographic information, laboratory tests and imaging results, chronic drug therapy, and hospital admissions. All subjects of Ethiopian ancestry (either born in Ethiopia or whose both parents were born in Ethiopia) aged 20 years or older on 12.31.2007, were included in the cohort (n = 8,398). A sample of 15,977 non-Ethiopian Jews (approximately twice as big as the Ethiopian sample) was randomly selected and group-matched for 10-year age group, sex and community clinic. Matching for community clinic was done to reduce the imbalance in socioeconomic status between Ethiopian and non-Ethiopian subjects. Subjects who died within the first year after inclusion (until 12.31.2008) were excluded from the cohort, in order to eliminate an effect of severe morbidity on blood glucose levels and body mass index (BMI).

Most of the Ethiopian Jews were immigrants (N = 7,994, 95.2%), and only 404 were born in Israel. Within the non-Ethiopian Jewish group; 10,828 subjects were born in Israel, 1,541 immigrated to Israel from the FSU after 1980, and the remaining 3,608 subjects either immigrated to Israel before 1980 or immigrated after 1980 but not from the FSU. To study the effect of immigration, comparisons were also made between the Ethiopian immigrants, FSU immigrants and non-Ethiopian Jews born in Israel subgroups. The subgroup of 3,608 non-Ethiopian Jews who emigrated before 1980, and who have lived for several decades in Israel, are not considered further as a separate subgroup in this paper. We used the data collected from 01.01.2007 until 12.31.2011. Prevalent diabetes was defined as diagnosis of diabetes or purchases of 3 or more hypoglycemic drug prescriptions by 12.31.2007. Incident diabetes was defined as a physician diagnosis of diabetes, purchases of 3 or more hypoglycemic drug prescriptions, or at least two raised values tested within a period of 12 months: of either a fasting glucose (≥126mg/dl) or a post-75gr oral glucose load plasma test (≥200mg/dl) or a HbA1c (≥6.5%, i.e. ≥48 mmol/mol)[17], between 01.01.2008 and 12.31.2011, among subjects who were not diagnosed as diabetic prior to 12.31.2007.

### Ethical considerations

The CHS institutional ethics committee approved the study protocol. In accordance with the Israeli Ministry of Health regulations, informed consent was not required, because all identifying information was removed prior to data analysis.

### Statistical analysis

The comparisons of baseline characteristics between Ethiopian Jews and non-Ethiopian Jews and between subgroups were carried out using the Student’s t-test for continuous variables and the chi square test for discrete variables. Age and sex data were complete. The amount of missing data for MetS variables was: BMI 17%, high-density lipoprotein cholesterol (HDL-C) 19%, triglycerides 18%, and blood pressure 10%. The proportions of missingness were very similar across the main groups and also across the subgroups. For example, for BMI the proportions of missingness were: non-Ethiopians 17%; Ethiopians 16%; subjects born in Israel 18%; FSU 16%; Ethiopian immigrants 16%, and for blood pressure they were: non-Ethiopians 11%; Ethiopians 8%; subjects born in Israel 12%; FSU 11%; Ethiopian immigrants 8%. We therefore judged that no statistical adjustment for missingness was necessary in the comparison of groups. The 2007 prevalence rates and weighted annual incidence rates of diabetes averaged over the period 2008–11 were calculated, employing the direct standardization method to adjust for differences in age and sex distributions, using the total Jewish population of Israel for those years (2007 for prevalence and 2008–11 for incidence) as the reference population group. Age-specific rates used for age standardization were in 10-year strata (e.g. 20–29 etc.) [18]. The formula for the calculation of the weighted incidence rates is described in S1 Appendix.

Cox proportional hazards models were used to analyze the association between ethnicity and diabetes risk and to estimate hazard ratios, using age as the time scale and non-Ethiopian Jews as the reference group. For subgroup analysis, the Jews born in Israel served as the reference group. Because the risk of diabetes increases with age, and in order to preserve the proportional hazards assumption, a variable classifying the cohort into 3 categories according to the person’s age at the start of follow up (20-49yrs, 50-59yrs and ≥60yrs) was included in the model. The hazard ratios for the ethnic groups were adjusted for sex, either alone or together with the first values recorded of the specific MetS components: triglycerides, BMI, HDL-C and systolic blood pressure (SBP). The MetS components were included in the models grouped in quartiles. An extra category was included for missing values. Interactions of each of the MetS components with ethnic group were analyzed.

We compared the average BMI values between subjects with and without diabetes according to age and ethnic-specific subgroups, using ANOVA and t-test statistics. For this analysis, diabetic subjects whose first BMI was recorded after developing diabetes, were excluded. Using multiple linear regression models, BMI was regressed on sex, diabetes status, ethnic group and the interaction between diabetes status and ethnic group, within a given age group.

## Results

Selected characteristics of the Ethiopian and non-Ethiopian Jewish main groups, and for the subgroups, are presented in Table 1. The mean ages of the non-Ethiopian and Ethiopian groups on 1.1.2008 were 40.5y and 39.4y respectively, while the born in Israel subgroup was much younger, 34.0y.

**Table 1:** Characteristics of the study cohort.

Main groups			Subgroups		
			Non-Ethiopian		Ethiopian
Variables	Non-Ethiopian N = 15,977	Ethiopian Jews N = 8,398	Born in Israel N = 10,828	FSU immigrants N = 1,541	Ethiopian immigrants N = 7,994
Age, years (mean ±SD)	40.5 ±18	39.4±17	34.0±12	40.0±16	40.3±17
Males (%)	50	48	51	46	49
BMI, kg/m^2^,(mean ±SD)	26.0± 5	23.6±4	25.4±5	26.5±6	23.7±4
HDL-C, mg/dl, (mean ±SD)	49±13	50±12	49±13	51±13	50±12
Triglycerides, mg/dl median (IQR)	105 (75–149)	96 (70–137)	101 (72–145)	105 (74–148)	97 (71–138)
SBP, mmHg, (mean ±SD)	120±14	121±14	117±13	122±14	121±14
DBP, mmHg, (mean ±SD)	73± 8	74±8	73±8	75± 7	74±8

Abbreviations: DBP: diastolic blood pressure; IQR: interquartile range;

### Diabetes prevalence and incidence

The overall age and sex-adjusted diabetes prevalence rates were similar among Ethiopian and non-Ethiopian Jews, 10.4 (9.6–11.2) and 10.3)9.7–10.9), respectively. However, the age-specific rates were significantly higher among Ethiopian Jews in the <50 years age group, 3.6 (3.1–4.1) vs. 2.7 (2.3–3.0) (p<0.01) (Table 2).

**Table 2:** Standardized and age-specific prevalence and weighted average annual incidence per 100 persons of diabetes (95% confidence interval)* over the period of 2008–11.

	Total		Men		Women	
Age (in years)	Non-Ethiopian Jews	Ethiopian Jews	Non-Ethiopian Jews	Ethiopian Jews	Non-Ethiopian Jews	Ethiopian Jews
All ages						
Prevalence	10.3 (9.7–10.9)	10.4 (9.6–11.2)	10.7 (9.9–11.6)	9.2 (8.2–10.3)	9.7 (8.9–10.6)	11.6 (10.4–12.9)
Incidence	1.8 (1.7–1.9)	2.1 (1.9–2.3)	2.0 (1.8–2.2)	2.1 (1.9–2.4)	1.6 (1.5–1.8)	2.2 (1.8–2.4)
20–49						
Prevalence	2.7 (2.3–3.0)	3.6 (3.1–4.1)	2.7 (2.3–3.2)	3.4 (2.7–4.2)	2.6 (2.1–3.1)	3.9 (3.1–4.7)
Incidence	0.5 (0.4–0.6)	0.9 (0.8–1.0)	0.6 (0.5–0.7)	1.0 (0.8–1.2)	0.4 (0.3–0.5)	0.8 (0.7–1.0)
50–59						
Prevalence	15.0 (13.2–16.9)	17.1 (14.5–20.1)	17.6 (15.0–20.6)	16.4 (12.8–20.9)	12.4 (10.2–14.8)	17.7 (14.0–21.9)
Incidence	2.6 (2.2–3.0)	3.6 (3.0–4.3)	2.8 (2.2–3.4)	4.1 (3.1–5.1)	2.4 (1.9–2.9)	3.2 (2.3–4.0)
60+						
Prevalence	29.8 (27.7–31.4)	26.0 (23.3–29.0)	32.4 (29.3–35.7)	23.3 (19.6–27.4)	27.2 (24.5–30.2)	28.4 (24.3–32.9)
Incidence	4.6 (4.1–5.0)	4.0 (3.4–4.7)	5.4 (4.6–6.1)	3.8 (3.0–4.7)	3.9 (3.3–4.4)	4.3 (3.4–5.2)

Age-specific prevalence and incidence rates were calculated for 10 years intervals (e.g. 20–29, etc.) and then grouped into 3 categories according to the person’s age at the start of follow up.

* Estimates are age- and gender- (where appropriate) standardized to the total Jewish population.

The standardized diabetes annual incidence rates per 100 persons (95% confidence interval in parentheses) were higher among Ethiopian Jews: 2.1 (1.9–2.3) than among non-Ethiopian Jews 1.8 (1.7–1.9), mainly among women. However, the incidence rates among men did not differ (Table 2). The largest differences in diabetes incidence rates were observed among people younger than 50 years: 0.9 (0.8–1.0) in the Ethiopian Jews as compared to 0.5 (0.4–0.6) among non-Ethiopian Jews, for both men and women. In the 50 to 59 years age group, diabetes incidence was somewhat higher in Ethiopian Jews 3.6 (3.0–4.3) compared to non-Ethiopian Jews 2.6 (2.2–3.0). However, the age-specific prevalence rates in the oldest group (≥60 years) were significantly lower among Ethiopian men with a trend to a lower incidence (not statistically significant). Ethiopian women were not different from non-Ethiopians in this respect.

### Diabetes risk in Ethiopian and non-Ethiopian immigrants and Jews born in Israel adjusted for the various MetS components

Adult Ethiopian Jews had higher diabetes risk until the age of 60 years, especially among the youngest age group (<50 years) (Table 3). Adjustment for triglycerides, HDL-C and SBP did not substantially change the point estimates of diabetes risk. However, BMI-adjustment in fact increased the HRs in Ethiopian Jews, mainly in the youngest age group from 1.81 (95%CI: 1.50–2.17) to 2.31 (95% CI: 1.91–2.79), but also in the other age groups. The risk for diabetes in elderly Ethiopian Jews (≥60 years) was not very different from that of non-Ethiopian Jews, (HR 0.89 (95%CI: 0.73–1.08)). To further analyze the impact of ethnicity vs. migration on the actual risk of developing diabetes, we compared two different ethnic groups who immigrated to Israel after 1980 (e.g. Ethiopian and FSU) to Jews born in Israel. Whereas Ethiopian immigrants had a high risk compared to those born in Israel among those <50y (HR 1.8 (95%CI: 1.5–2.2)), no such increased risk was seen among FSU immigrants (HR 0.9 (95%CI: 0.6–1.4)) (Table 3).

**Table 3:** Cox proportional hazard ratios (95% confidence interval) for incident diabetes by ethnicity, age, sex and components of the metabolic syndrome*.

Age (years)	M	Sex	Sex and Triglycerides	Sex and BMI	Sex and HDL-C	Sex and SBP
**20–49**	Non-Ethiopian Jews (reference), n = 11,244	1.0	1.0	1.0	1.0	1.0
	EJ n = 6,029	1.81 (1.50–2.17)	1.95 (1.62–2.35)	2.31 (1.91–2.79)	1.84 (1.53–2.22)	1.72 (1.43–2.07)
**50–59**	Non-Ethiopian Jews (reference) n = 1,533	1.0	1.0	1.0	1.0	1.0
	EJ n = 725	1.36 (1.07–1.73)	1.34 (1.05–1.70)	1.55 (1.22–1.98)	1.30 (1.03–1.66)	1.34 (1.05–1.70)
**60 and up**	Non-Ethiopian Jews (reference) n = 1,906	1.0	1.0	1.0	1.0	1.0
	EJ n = 968	0.89 (0.73–1.08)	0.87 (0.72–1.07)	1.02 (0.83–1.24)	0.87 (0.71–1.06)	0.90 (0.73–1.09)
	**Sub-group analysis**					
**20–49**	Born in Israel (reference) n = 9,172	1.0	1.0	1.0	1.0	1.0
	Ethiopian immigrants n = 5,638	1.8 (1.5–2.2)	2.0 (1.6–2.4)	2.4 (1.9–2.9)	1.9 (1.5–2.3)	1.7 (1.4–2.1)
	FSU immigrants n = 1,106	0.9 (0.6–1.4)	1.0 (0.6–1.5)	0.9 (0.6–1.4)	1.0 (0.7–1.6)	0.9 (0.6–1.4)
**50–59**	Born in Israel (reference) n = 901	1.0	1.0	1.0	1.0	1.0
	Ethiopian immigrants n = 719	1.3 (1.0–1.6)	1.3 (1.0–1.7)	1.5 (1.1–1.9)	1.2 (0.9–1.6)	1.3 (1.0–1.6)
	FSU immigrants n = 152	0.7 (0.4–1.3)	0.8 (0.4–1.3)	0.7 (0.4–1.2)	0.8 (0.5–1.4)	0.7 (0.4–1.2)
**60 and up**	Born in Israel (reference) n = 301	1.0	1.0	1.0	1.0	1.0
	Ethiopian immigrants n = 968	0.8 (0.6–1.1)	0.8 (0.5–1.1)	1.0 (0.7–1.4)	0.8 (0.5–1.1)	0.8 (0.6–1.1)
	FSU immigrants n = 185	0.9 (0.5–1.4)	0.9 (0.5–1.4)	0.8 (0.5–1.3)	0.9 (0.6–1.5)	0.8 (0.5–1.3)

* Table 3 includes data on subjects not diagnosed with diabetes until 1/1/2008.

### BMI according to ethnicity and diabetes status

Across all age and ethnic subgroups, subjects who developed diabetes during follow-up had on average a higher baseline BMI than subjects who remained non-diabetic. Ethiopian immigrants, either with or without diabetes, had the lowest mean BMI (p<0.0001) (Table 4). Moreover, in the youngest age group (<50y), the mean BMI difference between diabetic and non-diabetic persons was significantly smaller among Ethiopian immigrants, 3.0 kg/m^2^, compared to non-Ethiopian Jews born in Israel, 5.3 kg/m^2^ (p < .001). In fact, the mean BMI values in those who developed incident diabetes among Ethiopian immigrants were similar to the mean BMI values of subjects without diabetes among FSU immigrants and Jews born in Israel. A subsequent multiple regression analysis which examined the effects of sex, ethnic subgroup, diabetes status and the interaction between them on BMI, revealed a statistically significant interaction between Ethiopian ethnicity and diabetes status (p = 0.0001).

**Table 4:** BMI among patients with incident diabetes and healthy controls by ethnicity and age*.

Population	<50 years			50–59 years			60+ years		
	Non-diabetic	Diabetic	p	Non-diabetic	Diabetic	p	Non-diabetic	Diabetic	p
Jews born in Israel (n = 10374)	(n = 7290) 24.8 ± 5.0	(n = 131) 30.1 ± 5.6	<0.0001	(n = 664) 27.0 ± 4.8	(n = 87) 28.7 ± 5.6	0.006	(n = 212) 26.7 ± 4.3	(n = 42) 28.1 ± 4.1	0.046
Ethiopian Immigrants (n = 7325)	(n = 4387)**22.9 ± 3.8	(n = 169)* 25.9 ± 4.2	<0.0001	(n = 525)** 25.0 ± 3.8	(n = 88) 27.3 ± 4.3	<0.0001	(n = 706)**24.3 ± 3.8	(n = 116)**25.5 ± 3.5	0.002
FSU immigrants (n = 1443)	(n = 881) 25.3 ± 4.9	(n = 16) 29.0 ± 5.8	0.003	(n = 122)** 28.4 ± 5.8	(n = 7) 31.0 ± 7.6	NS	(n = 135)** 28.1 ± 5.1	(n = 21)** 32.3 ± 6.2	0.0008

* The first BMI recorded during the study period was used for this analysis. Diabetic subjects whose BMI values were missing or whose first BMI value was recorded after developing diabetes were excluded; thus BMI was available for 81%, 82%, and 82% non-Ethiopian Jews born in Israel, Ethiopian immigrants and FSU immigrants, respectively. BMI results are presented as mean ±SD,

** statistically significant difference comparisons within age and diabetes status (Yes/No) subgroups, where non-Ethiopian Jews born in Israel was the reference category, P <0.001.

## Discussion

This historical cohort study of the Jewish communities in Israel highlights the emerging risk of diabetes among Ethiopian immigrants to Israel. This disease has dramatically evolved in just 2–3 decades from close to non-existent to one of high prevalence, compared to Jews born in Israel, among subjects younger than 50 years, and one of similar prevalence to Jews born in Israel among those over 60-years-old. Of note is that, while age-specific prevalence rates in the youngest age group are higher among all Ethiopian Jews, in other age groups apparently, only women are affected. However, the incidence rates in the 20-49y age group are higher for both men and women. Possible explanations could be an earlier increase in diabetes incidence among Ethiopian women than men or less frequent medical visits among younger Ethiopian men than women, leading to less frequent diagnosis.

Research conducted in sub-Saharan Africa between the 1960s and early 1980s showed that diabetes prevalence was generally less than 1% [2,5]. More recent studies showed low diabetes prevalence in several rural communities [19], with moderate rates recorded in both rural and urban populations (Tanzania 5.7%, Republic of Guinea 6.7%) [19–20]. The highest rates were reported among internal rural-to-urban migrants and in communities of Africans living abroad [21–22]. The prevalence of diabetes in genetically similar populations of African ancestry is 0.8% within rural Cameroon, 2.0% in urban Cameroon, 8.5% in Jamaica, and 14.6% in England [22]. Most of the studies from sub-Saharan Africa are cross-sectional by design. Thus information regarding diabetes incidence is almost non-existent [23].

The trends in diabetes prevalence and incidence in our cohort differed by age. The largest difference between Ethiopian and non-Ethiopian Jews was observed in those younger than 50 years of age, while only small differences were seen among those 60 years or older. Ethnic differences in Type 2 diabetes risk and age at presentation have been previously reported in Israel among Arabs and Jews [24]. Chiu et al. found that compared to the Caucasian population of Ontario Canada, diabetes developed earlier in residents of non-European origin, both immigrants and those born in Canada; an average of nine years earlier among South Asians, three years earlier among Chinese and one year earlier in African Canadians [25].

There are several possible explanations why the excess of diabetes risk was not observed among Ethiopian immigrants who were 60 years or older. Healthy migration selection may be a plausible explanation, meaning that only the strong and healthy elderly subjects survived the hardships of the long arduous journey of the Ethiopian immigration to Israel [26]. The impact of such selection was possibly less prominent in young people. However, there are no solid data on mortality during the migration process that can either confirm or reject this explanation. Second, adoption of a Western lifestyle, which leads to the increased risk of diabetes, may be more common in young adults than in the elderly, who are less apt to change their lifestyle. Support for this explanation comes from the observation that communities who maintained their traditional lifestyle were less likely to develop diabetes [6, 27]. Yet, information on lifestyle habits in Ethiopian Israelis in different age groups is still not available to validate this assumption. The age of the Ethiopian immigrants in most cases was not based upon birth certificates, so a tendency to overestimate people's ages at the time of immigration, in the case of older-appearing individuals, can play some role. However, many of the other immigrants (before 1980) also did not have birth certificates and share the same bias with the Ethiopian immigrants. Since the HR for diabetes in the main groups and in the subgroups is very similar, we do not think this factor is of major importance. Finally, it is possible that the detrimental physiologic effects of changes in diet and physical activity and of exposure to endocrine disruptors in the new Israeli environment differ by age.

While non-Ethiopian males had a higher diabetes incidence than females, no such gender effect was observed among the Ethiopian Jews. Gender differences in diabetes risk with male predominance have been reported in several ethnic groups, including those of European and Korean ancestry [28–29]. A possible explanation relies on the observation that males are generally more insulin resistant than females [30], thus requiring a smaller weight gain than females for them to develop diabetes [31]. However, in sub-Saharan Africa, prevalence of diabetes and obesity are both higher among females [32].

The excess risk for diabetes found among young Ethiopian Jews was not explained by increased prevalence of abnormal levels of triglycerides, HDL-C or systolic blood pressure. In fact, we found that Ethiopian Jews develop diabetes at significantly lower BMI values and adjustment for BMI yielded a further increase in the hazard ratio for diabetes. Among Ethiopian immigrants, we found that the BMI difference between those who developed diabetes and those who remained non-diabetic was significantly smaller compared to both FSU immigrants and born in Israel non-Ethiopians. Lower BMI values at diabetes diagnosis were reported also in Asian Americans [25]. Recent findings from rural sub-Saharan Africa suggest that the optimal cut-offs of BMI to identify cardio-metabolic risk (hypertension, diabetes and dyslipidaemia), ranged from ≥23kg/m^2^ to ≥25kg/m^2^ for men and from ≥24kg/m^2^ to ≥26kg/m^2^ for women, concurring with our results [5, 33]. Wai et al [34] reported that waist circumference is the best predictor of cardiovascular disease risk among urban Ethiopian adults.

The excess risk for diabetes found among young Ethiopian immigrants was not observed among Caucasian immigrants from the FSU. This is in contrast to the higher prevalence of diabetes previously reported among FSU immigrants to Israel and the USA [16, 35]. Possible explanations for this difference are a substantially better lifestyle and medical therapy adherence of FSU immigrants to Israel as compared to Jews born in Israel [16], a higher socio-economic status, or selection effects. Our data on FSU immigrants argue against the assumption that migration to Israel and acculturation stress per se underlies the increased diabetes risk among Ethiopian Jews, and may indicate a greater genetic susceptibility or thrifty phenotypic alterations secondary to under-nutrition in Ethiopia [36–37], which become evident after a change in traditional lifestyle and environment, followed by weight gain.

The data in our study were collected for clinical management purposes and are limited to observations made during clinic visits. However, since access to healthcare services in Israel is universal, all study subjects had free access to primary practitioners and laboratory testing, thus reducing a potential information bias. One limitation is that the information provided in the records was not detailed enough to identify the type of diabetes. Because the Ethiopian immigration took place over a short period, less than a decade, there was rather little variation in the length of residency in Israel in this cohort, so we could not examine the effect of length of stay within Israel on diabetes risk. A longer follow-up period is needed to study this effect. Our database did not enable adjustment for individual socioeconomic status or educational level, factors known to affect the risk for developing diabetes. We partially controlled for socioeconomic status by selecting the non-Ethiopian cohort from those living in the same residential areas as Ethiopians. However, this design of the study cohort may have also affected our findings. A previous study in Israeli Jewish adults of higher socioeconomic level [16] showed lower overall diabetes prevalence (7.1% versus 10.3% found in our study). Consequently, our results may underestimate the difference between Ethiopian and non-Ethiopian Jews. We also did not have data on body fat distribution, as measured by waist circumference or waist-to-hip ratio.

The major strengths of this study are the availability of longitudinal data and the large sample size enabling more precise estimates of diabetes risk and underlying population risk differences.

We have found that Ethiopian ethnicity is an independent predictor of diabetes risk that is age dependent and BMI sensitive. Increased diabetes risk at a young age is a challenge to patients, the health care system and society. Chronic disease at a young age affects productivity and the individual’s socio-economic status. In addition, earlier onset of diabetes increases the risk of developing diabetes-related complications in later life. Moreover, diabetes among young women during their years of fertility may adversely affect their offspring. Ethiopian Israelis, especially women and those who are younger, should be prioritized in diabetes screening and prevention programs. In addition, we suggest that the BMI cut-offs used for diabetes screening need to be redefined for Ethiopian Israelis. Additional studies are required to decipher the role of fetal and early life nutrition, lifestyle habits and visceral adiposity in the pathogenesis of diabetes in Ethiopian immigrants, and to confirm and explore the seemingly different effects of ethnicity in older Ethiopian immigrants.

## Supporting Information

S1 Appendix (DOCX)
