# Supplementary material for: Diabetes among Ethiopian Immigrants to Israel: Exploring the Effects of Migration and Ethnicity on Diabetes Risk
Source: PLoS One. 2016 Jun 14;11(6):e0157354. doi: 10.1371/journal.pone.0157354 (PMC4907509; doi:10.1371/journal.pone.0157354)
Supplement: S1 Appendix — (DOCX) [file pone.0157354.s001.docx]

### Supporting Information

S1 Supplement

For any year j and m age groups the ASR will be;

$$\sum_{i=1}^{m} \frac{r_{ij}}{n_{ij}}\times\frac{N_{ij}}{N_{j}}$$

where $r_{ij}$ = the number of diabetes cases in age group i of the subpopulation in year j,

$n_{ij}$= the number of persons in age group i of the subpopulation in year j,

$N_{ij}$= the number of persons in age group i of the total population in year j, and

$N_{j}$= the total number of persons in the total population in year j.

And thus the weighted ASR for 4 years and 16 age groups will be;

$$\sum_{i=1}^{16} \left[ \frac{\sum_{j=1}^{4} r_{ij}}{\sum_{j=1}^{4} n_{ij}}\times\frac{\sum_{j=1}^{4} N_{ij}}{\sum_{j=1}^{4} N_{j}} \right]$$

where;$\sum_{j=1}^{4} r_{ij}=r_{i}$

$\sum_{j=1}^{4} n_{ij}=n_{i}$

and where the variance is given by;

$$\sum_{i=1}^{16} \left[ \frac{r_{i}\left( n_{i} \right.-\left. r_{i} \right)}{n_{i}^{3}}\times\left\{ \frac{\sum_{j=1}^{4} N_{ij}}{\sum_{j=1}^{4} N_{j}} \right\}^{2} \right]$$
